# Newborn Hearing Screening: the relation between bathing and the retesting rate

**DOI:** 10.1016/S1808-8694(15)30571-1

**Published:** 2015-10-19

**Authors:** Tatiana Redeschi Marques, Patrícia Christina Mendes, Cristiane Franceschi Pineroli Bochnia, Lilian Cássia Bornia Jacob, Simone Mariotto Roggia, Jair Mendes Marques

**Affiliations:** 1 Specialist in Clinical Audiology from the University of Tuiuti in Paraná, Speech therapist at Detecta Clinic in Curitiba - PR.; 2 Specialist in Clinical Audiology from the University of Tuiuti in Paraná, Speech Therapist at Detecta Clinic in Curitiba - PR.; 3 M.Sc in Communication Disorders from the University of Tuiuti in Paraná, Speech Therapist at Detecta Clinic in Curitiba - PR.; 4 PhD in Human Communication Disorders at USP - Bauru, Speech Therapist. Professor at the Post-Graduate Program on Communication Disorders and Speech Therapy Course at the University of Tuiuti in Paraná.; 5 PhD in Sciences- Experimental Speech Therapist at USP – São Paulo, Speech Therapist. Professor at the Post-Graduate Program on Communication Disorders at the University of Tuiuti in Paraná.; 6 PhD in Geodesic Sciences, Professor at the Post-Graduate Program on Communication Disorders and on the Speech Therapy Course at the University of Tuiuti in Paraná.

**Keywords:** hearing, newborn, mass screening, neonatal screening

## Abstract

One of the goals of a Newborn Hearing Screening Program (NHSP) is to have a low retesting rate.

**Aim:**

To investigate the association between the retesting rate and the bath of newborn babies. Study design: cross-sectional contemporary cohort.

**Materials and Methods:**

Transient otoacoustic emissions (TOE) results have been compared to the information received from the nurse's aide who bathed the babies on the day of the test (373 newborns), and the time interval between the TOE study and the last bath (350 newborns).

**Results:**

Significant statistical differences were found in relation to the percentage of retesting when babies were bathed by certain nurse's aides. On the other hand, the percentage of retesting decreased significantly when the time interval between the last bath and the TOE study was longer than 7 hours and 50 minutes.

**Conclusion:**

Moisture of the external acoustic meatus, caused by inappropriate protection against water during bath, and the short interval between the bath and the ETOE study, could be considered as possible factors causing retesting in NHSP programs.

## INTRODUCTION

Hearing deficiency is a serious problem in public health. However, since it is not evident in the first months of life, this deficiency may have a late diagnosis, and as a consequence, it may cause damages to speech development and to the overall development of the child.

American Academy of Pediatrics^1^ estimates an incidence of hearing deficiency in three out of 1.000 newly born children. In our country, since it is not common to have Universal Newborn Hearing Screening (UNHS) in all maternity hospitals, it is difficult to have a national incidence. However, the only epidemiologic study done in Brazil on hearing loss prevalence had the result of 6.8% prevalence of disabling hearing loss^2^. Hearing deficiency prevalence is greater than phenylketonuria (1 in 10,000), hypothyroidism (2.5 in 10,000) and anemia (2 in 10.000)^3^, easily identified with the Heel Stick Test.

The rate of children born alive reported in Datasus website indicates the birth of 3,026,548 babies in 2004 in Brazil^4^. Based on this data and taking into account the incidence of three out of 1,000 newborns, we can deduce that 25 children are born daily in Brazil with hearing alterations.

According to the Joint Committee of Infant Hearing^5^, the ideal situation would be to diagnose hearing deficiency before three months of age and to start therapeutic intervention before six months of age. A study done at the University of Colorado in the United States, by Yoshinaga-Itano et al.6 showed that children with hearing deficiency identified before sixth months of age, had a significant difference in speech when compared to children who had a hearing problem identified after they were six months old.

Despite the importance of early intervention for the development of those children with hearing deficiency, the average age at diagnosis of hearing deficiency in Brazil is late. Nóbrega7, evaluated 442 subjects with hearing deficiency from 1990 to 1994, and from 1994 to 2000, revealed that even with the increased information available for the population and a higher level of medical knowledge on target exams, considering the second period studied, this average age did not show significant statistic changes, remaining at around two years old. In our clinical practice, we see that hearing alterations are commonly identified by speech delay generally at 18 to 24 months old.

In order to prevent this situation, UNHS, that is to say, to screen the hearing of all babies born at maternity hospitals is being done in some Brazilian maternity hospitals, as it is done in the United States and Europe.

The importance of programs for early identification of hearing deficiency, in which all newborn babies must be screened, is stressed by the widely disseminated data that 50% of patients with hearing difficulties do not show risk factors for hearing deficiency^8^, ^9^. Thus, in such situation 50% of hearing loss cases would not be identified in the absence of a universal screening program.

However, it is noted that despite UNHS being one of the most important tools in early identification programs for hearing disorders, its functionality depends directly on the criteria used to do it.

The National Institutes of Health, in 1993^10^ and the Joint Committee on Infant Hearing, in 1994^8^, made recommendations, suggesting the use of Evoked Otoacoustic Emissions (EOE) or of Brain Stem Hearing Potential as the best methods for Newborn Hearing Screening (NHS). Since then, the use of these methods in early identification programs for hearing loss started to gain momentum also here in Brazil.

In the last survey done by the Newborn Hearing Screening Supporting Group in 2005, it was seen that there were 237 places in 22 states developing NHS Programs with an objective methodology (EOE and/or Hearing Potential of Encephalic Trunk)11 in Brazil. At the International Conference Beyond Newborn Hearing Screening, that took place in Como, Italy in 2004, Chapchap and Ribeiro reported that out of the 192 places that at the time had NHS Programs in Brazil, 61% used transient evoked otoacoustic emissions (TEOAE), 24% evoked otoacoustic emissions- distortion product and 15% Brain Stem Hearing Potential^12^.

With objective exams, NHS Programs became feasible. However, there are difficulties. In the study of Evoked Otoacoustic Emissions for example, some factors may distort the result, such as professional training when placing the probe; excessive noise during the exam; baby´s condition at the moment of the exam, that is to say, if he/she is asleep or awake, hearing screening test within the first 24 hours of life, a period that increases the possibility of vernix in the external acoustic meatus^13^.

Despite confirmation of above mentioned factors, there is not enough literature regarding the difficulties found with Evoked Otoacoustic Emissions specifications at NHS. A difficulty that was mentioned more frequently is the presence of vernix. Chang et al.^14^, for example, studied Evoked Otoacoustic Emission results in 41 newborns with an average age of 43 hours old and they observed that after the removal of amniotic fluid and vernix, the positive results went from 76% to 91%.

In order to establish the final result, without interference from the possible presence of vernix, re-testing is normally indicated no longer than 30 days of life. However, we also observed in our practice that the required time for re-test can cause anxiety in the family as a consequence and it can also create a lasting doubt for speech therapists, due to the failure of patients to come back to be re-tested. Thus, the recommendation resulting from this statement is that we should try to achieve a reduction of re-tests.

Vernix is defined as a lipid rich substance that covers the fetus skin and it is also present in the newborn skin^15^. It has an important role in the protection against infections,^15^ and inside the womb it works as a hydrophobic barrier to protect and waterproof developing skin against the surrounding amniotic fluid^16^.

Since vernix is a fatty substance which does not mix up with water, it is believed that there may be a greater difficulty of water absorption when there is vernix. It is supposed that this may explain our practical observation that after bath, it is often more difficult to capture TEOAEs, possibly due to humidity in the external acoustic meatus.

Another issue that called our attention in our practice was that when we did the same amount of exams in alternate days, at the same maternity hospital, the number of re-tests showed a sensitive alteration, for example, on one day, in 10 screenings, not even one re-test whereas on the following day, with the same amount of exams, there were three or four re-tests.

The following hypothesis resulted from our practical observation:

- re-testing rate could vary depending on the person responsible for the baby´s bath, due to the way and/or the experience of the assistant nurse who allows water into the external acoustic meatus during bath;
- the re-testing rate could vary depending on the time passed between the last bath and the Evoked Otoacoustic Emissions test, because with moist external acoustic meatus, the failure in the exam seemed to be even more frequent, mainly with the presence of vernix.

Taking into account these hypothesis and considering the importance of trying to reduce the number of re-tests on the EOE study at NHS, this research aimed to investigate the relationship between re-testing rate and the newborn bath, at a NHS program, considering the professional responsible for the bath and the time passed between the TEOAE study and the last bath.

## MATERIAL AND METHOD

This research was approved by the Research Ethics Committee at the University where we did the study, authorization no 017/2004. Following current standards on ethics on research with human beings, all participants (both individuals and/or those responsible for the babies) of this study agreed on taking part and they received information on the project and signed an Informed Consent.

Data were gathered at a maternity hospital in Curitiba, where there is a Hearing Deficiency Early Identification Program. This Program started in 1996 and its methodology uses TEOAE in newborn children. All screenings are done within the first 24 hours or more of life, preferably closer to the hospital discharge (approximately 48 hours old), in a big room with the baby in the adequate state and it is done by speech therapists with a large experience on NHS. Thus, the possible re-tests resulting from the lack of training of the professional in placing the probe, excessive noise during the exam, an inadequate state of the baby at the moment of the test and hearing screening before the baby is 24 hours old were avoided.

After the test, when the baby does not pass the exam, a new test is scheduled in 30 days. If TEOAE are still altered, the baby is sent for a complete audiological evaluation for diagnosis confirmation.

In the daily routine of maternity hospitals, when babies are born they are kept in a warmed crib for about two hours and then they received the first bath. Assistant nurses are responsible for baths and they use a special technique to place the fingers pressing the tragus to prevent water from coming in.

We started gathering data for this study in March 2004 and we finished in June the same year. Despite the fact hearing screening is optional, we screened 130 babies per month for each 200 born babies on average, making up a total of 538 babies on NHS during the period of the study. However, the study of the causes of this research was different according to the variable we studied, taking into account the amount of information that can be obtained regarding each baby.

The first analysis done tried to check the correlation between the assistant nurse who gave the bath just before the TEOAE test and the of re-tests. On the second analysis, we looked at a possible correlation between re-testing rate and the time span between the last bath and the TEOAE test.

Data related to the assistant nurse responsible for the last bath before the test and to the time span between the bath and the TEOAE study were gathered with the help from the team of assistant nurses at the nursery, who filled in a form to record all this information (APPENDIX A).

**Appendix A:** Analysis of the number of re-tests according to the time span between the bath on the day of the exam and the OAE test.

Date	Mother´s name	Room	Assistant nurse who bathed the baby	Time of bath	Remark
					
					
					

Considering that not all assistant nurses filled in all necessary data for all babies, the number of babies we studied was different according to the variable studied, that is to say, 373 babies for the variable of the assistant nurse who gave the last bath and 350 babies for the variable of the time span between the last bath and the TEOAE. Thus, sample inclusion criteria were to have forms completely filled out by the nursing team and the lack of risk factors for hearing loss^5^.

For TEOAE test, we used the ILO Echocheck equipment by Otodynamics, and as stimulus we used a click in an intensity of 83 dBNPS, and the frequency range analyzed was between 1,600 and 3,600 Hz. For the normality standard the equipment allows us to choose the minimum values of the signal/noise ratio, as well as the response level of TEOAEs. In this study, the values adjusted as normality criteria for the presence of TEOAE (normal result of the exam) were the following: TEOAE amplitude and the signal/noise ratio as the same or above 6 dBNPS, according to what Román et al suggested.^17^.

The data obtained from the forms were analyzed together with TEOAE records.

Proportion Test was used in the statistic analysis with a significance level of 5%.

## RESULTS

Table 1 shows the results regarding the study of the number of re-tests according to the assistant nurse who gave the last bath just before the TEOAE test. It can be seen that 51 babies out of the 373 who had had the exam, failed at the first TEOAE test, that is to say, 13.67% of babies. In the re-test, only five babies remained with absent TEOAE and they were sent for a complete audiological evaluation. Later on, the lack of hearing disorders was confirmed.

**Table 1:** Analysis of the number of re-testing according to the assistant nurse who gave the bath on the examination day.

Nurse	Number of babies he/she bathed	Babies who passed the screening		Babies who needed a re-test		Normal Re-test		Amended Re-test	
		Relative Number (%)	Absolute Number	Relative Number (%)	Absolute Number	Relative Number (%)	Absolute Number	Relative Number (%)	Absolute Number
A	2	2	100	0	0	-	-	-	-
B	3	3	100	0	0	-	-	-	-
C	4	4	100	0	0	-	-	-	-
D	4	4	100	0	0	-	-	-	-
E	6	6	100	0	0	-	-	-	-
F	12	12	100	0	0	-	-	-	-
G	22	22	100	0	0	-	-	-	-
H	1	0	0	1	100	1	100	0	0
I	3	1	33,33	2	66,67	2	100	0	0
J	8	6	75	2	25	2	100	0	0
L	9	7	77,78	2	22,22	2	100	0	0
M	90	71	78,89	19	21,11	16	84,21	3	15,79
N	26	21	80,77	5	19,23	5	100	0	0
O	6	5	83,33	1	16,67	1	100	0	0
P	31	26	83,87	5	16,13	5	100	0	0
Q	13	11	84,62	2	15,38	2	100	0	0
U	14	12	85,71	2	14,29	1	50	1	50
V	24	21	87,5	3	12,5	2	66,67	1	33,33
X	10	9	90	1	10	1	100	0	0
Z	66	61	92,42	5	7,58	5	100	0	0
Y	19	18	94,74	1	5,26	1	100	0	0

TOTAL	373	322	86,33	51	13,67	46	90,2	5	9,8

Absolute number (absolute frequency) and relative number (relative frequency)

Taking into account that some assistant nurses only gave a few baths, we choose to do a statistical analysis including only those who gave at least 10 baths. Therefore, we used the Proportion Test with the aim of checking if the differences in percentages of re-tests obtained by these nursing assistants differed significantly. Considering a 5% significance level, we saw a statistical significant difference between the re-test percentages obtained by assistant nurses G and M (p value of 0.0202), G and N (p value of 0.0351) and M and Z (p value of 0.0231), suggesting that some assistant nurses may have produced more re-tests than others.

Another interesting data that may be obtained from looking at Table 1 data has to do with the fact that assistant nurses F and G do not have any re-test, despite having given a significant amount of baths.

Table 2 shows data on the analysis done regarding the possible correlation between re-testing rate and the time passed between the bath at the examination day and TEOAE test. Time division was done from the analysis of the notes taken in the form filled in by the nursing team (APPENDIX A), that is to say, we verified the distribution of newly born babies related to the different time intervals. Thus, their division was done taking into account the size of the sample in each category so that the number of newborn babies was similar to each one of them. Besides, we were concerned with not having too many time intervals with a small number of newborn babies, since this could compromise the statistical analysis.

**Table 2:** Analysis of the number of re-tests according to the time span between the bath on the day of the exam and the OAE test.

Bath time before the exam	Number of babies screened	Babies who passed the screening		Bebês que necessitaram de reteste		Reteste Normal		Reteste Alterado	
		Relative Number (%)	Absolute Number	Relative Number (%)	Absolute Number	Relative Number (%)	Absolute Number	Relative Number (%)	Absolute Number
Until 2 h	108	88	81,48	20	18,52	18	90	2	10
Between 2h1min and 5h50min	94	85	90,43	9	9,57	9	100	0	0
Between 5h51min until 7h50min	71	65	91,55	6	8,45	5	83,33	1	16,67
More than 7h51min	77	71	92,21	6	7,79	6	100	0	0

TOTAL	350	309	88,29	41	11,71	38	92,68	3	7,32

Absolute number (absolute frequency) and relative number (relative frequency)

It can be observed that 41 out of the 350 babies who had the TEOAE test done, had to be re-tested, that is to say 11.71% of them. Re-test results were as follows: 38 normal tests (92%) and three altered tests (8%). The three babies with altered re-tests were sent for diagnosis and they had normal results. As it can be seen on Table 2, the number of re-tests was reduced according to the increase in the time span between the bath and the TEOAE test.

From the results obtained and showed on Table 2, an analysis was used in the attempt of statistically checking the differences between the percentages of obtained re-tests according to the time passed between the last bath and the TEOAE test. As Table 3 shows, re-testing percentage was significantly reduced when the time passed between the last bath and the TEOAE test was more than 7 hours and 50 minutes.

**Table 3:** Proportion test to compare: bath-exam at a significance level of 5%.

Comparison	p	Result
Until 2 h x Between 2h1min and 5h50min	0,0740	Non significant
Until 2 h x Between 5h51min until 7h50min	0,0687	Non significant
Until 2 hours x More than 7h51min	0,0372	Significant

p = result of difference proportion test (significant with p < 0.05)

## DISCUSSION

One of the main concerns of professionals working with hearing deficiency early detection programs is related to re-testing rates^18^, since these re-tests may compromise the effectiveness of the program. Thus, the whole team involved in the program must work in order to increasingly reduce these rates.

The consequences of these re-tests can be many: anxiety of parents related to a possible confirmation of hearing deficiency, the baby does not show up for re-test and the consequent “loss” of the child for diagnosis, increased demand on the service flowchart of hospital/maternity/clinic, increased cost-effectiveness of the hearing screening program, among others.

Therefore, this study tried to investigate some factors that may contribute to a very important cause in the production of re-tests: moisture in the baby's external acoustic meatus at the hearing screening.

Among these factors, we can mention the work done by assistant nurses who bathe the baby on the day of the exam. We can state that some of them had re-testing percentages significantly different form others. It is believed that this confirmation may result from the more or less skills of these professionals to protect the baby's ear to avoid the water going into the ear at bath time. Furthermore, we also noticed that those assistant nurses who did not sent babies to be re-tested were the most experienced professionals of the sector, according to the information obtained from the head nurse. Thus, the findings obtained confirmed the hypothesis that re-testing rates can be influenced by the experience the assistant nurse has in bathing babies, avoiding or not water from coming into the external acoustic meatus.

Another result that confirms the hypothesis that water inside the babies’ external acoustic meatus may have caused the re-testing, has to do with the time span between the bath at the day of the exam and the TEOAE test. The results obtained on Table 2 showed that as the time passed between the bath and the TEOAE test increased, there was a reduction on the percentage of re-tests. The statistical difference on the re-testing percentage reduction was proven when the time between the last bath before the exam and the TEOAE test was more than 7 hours and 50 minutes (Table 2), suggesting that this time is safer for TEOAE tests. Thus, the results from the present investigation also confirm the hypothesis that says that re-testing rate may vary depending on the time span between the last bath and the TEOAE test.

Therefore, the results obtained on the analysis of the two variables (professional who gives the bath and waiting time between the last bath and TEOAE records) suggest that humidity on external acoustic meatus may be increasing the re-testing percentage of TEOAEs, considering that the variable experience of examiner, noise level at the room, exam made with less than 24 hours and the state of the baby at the moment of the exam were not considered for the methodology used.

It is believed that the persistent moisture at the external acoustic meatus may result from the presence of vernix, considering that it works as a hydrophobic barrier^16^ and therefore, posing some difficulty for water absorption.

We think it is important to point out that a possible cause for re-testing seen at this study was not found in any other study we reviewed. According to Maxon et al.^19^, there are publications that report the effect of some factors on the re-testing rates at NHS programs using TEOAE. According to these authors, the issues reported on these papers can be classified into the following categories: baby's time of life when the screening was done, conditions of the facilities where the screening was done, conditions of middle and external ear and re-testing rates adopted by the programs. Therefore, among the middle and external ear conditions, the authors only mention the presence of vernix, amniotic fluid or middle ear secretion.

Thus, the results obtained in this study suggest that we need to include specific guidance for nursing teams and parents into the protocols developed on NHS programs. These guidelines should include being extremely careful to avoid water coming into the baby's ears during bath. Besides, the results also suggest that the TEOAE test should be made with the highest possible time interval after the newborn's bath.

It is believed that if this care is taken, re-testing percentage may be considerably reduced and therefore it would be possible to reduce the cost-effectiveness of the hearing screening program, as well as the anxiety experienced by parents when receiving an altered result.

From this point of view, we stress that the success of an early identification program of hearing loss is related to the possibility of reducing false-positive results caused by the conditions of the external ear, among other factors.

## CONCLUSION

From the results obtained, we can conclude that moisture in the external acoustic meatus of babies, caused by a bath time too close to the study of otoacoustic emissions and by an inefficient protection of the external acoustic meatus regarding water penetrating the the ear at bath time, can be considered a possible factor that generates re-testing on NHS programs that use TEOAE as a hearing screening procedure.

The confirmation that the baby's bath at the day of the exam may influence the result of the TEOAE test, offers a very relevant information for NHS programs that have as one of their goals low re-testing percentages.
